# Physicochemical, antioxidant, antimicrobial, and in vitro cytotoxic activities of corn pollen protein hydrolysates obtained by different peptidases

**DOI:** 10.1002/fsn3.3252

**Published:** 2023-02-14

**Authors:** Amir Akbarmehr, Seyed Hadi Peighambardoust, Babak Ghanbarzadeh, Khashayar Sarabandi

**Affiliations:** ^1^ Department of Food Science, College of Agriculture University of Tabriz Tabriz Iran; ^2^ Department of Food Science & Technology, School of Medicine Zahedan University of Medical Sciences Zahedan Iran

**Keywords:** anticancer, antimicrobial, antioxidant, corn pollen protein, polypeptides

## Abstract

The applications of protein hydrolysates as food preservatives and nutraceutical ingredients have attracted much attention because of their beneficial effects. The interest in these ingredients has shifted toward their biological activities with benefits to human health. Bioactive peptides are known as antioxidant agents that could promote health‐promoting effects and prolong food shelf‐life beyond their basic nutritional value. Thus, the aim of this study was to investigate antioxidant, antimicrobial, and in vitro cytotoxic properties of corn pollen protein (CPP) hydrolysates obtained by different enzymes. Proteolytic activity in terms of degree of hydrolysis (DH) and SDS‐PAGE analysis was measured in pancreatin (H‐Pan), pepsin (H‐Pep), and trypsin (H‐Tri) hydrolysates. Amino acid composition, antioxidant and antimicrobial activities, and cytotoxicity of hydrolysates were evaluated. DH and SDS‐PAGE revealed higher proteolytic activity of pepsin compared to other enzymes. Amino acid analysis showed that the functional amino acids such as antioxidant types were most predominant in H‐Pep compared to two other samples. Antioxidant activity of hydrolysates was found to be affected by the type of enzyme and the concentration of hydrolysates. There was a significant difference (*p* < 0.05) between antioxidant activity of different hydrolysates. The highest antioxidant activity in terms of Trolox equivalent antioxidant capacity (0.23–2.75 mM), DPPH (33.3%–64.8%), and hydroxyl (33.7%–63.2%) radical scavenging activities, chelation of iron (33.2%–62.5%) and copper (30.2%–50.5%) metals, and total antioxidant activity (0.65–0.85) was obtained for H‐Pep followed by H‐Pan and H‐Tri samples. Antibacterial tests showed that pepsin‐hydrolyzed protein was not significantly (*P* > 0.05) effective against *E. coli* at any concentrations, however, it showed significant (*P* < 0.05) concentration‐dependent effect against *S. aureus* (with inhibition zones of 15–25 mm). Cytotoxicity results revealed that CPP, as a nonhydrolyzed protein, did not generally show antiproliferative activity, however, a significant (*P* < 0.05) ability of H‐Pep hydrolysate in decreasing HT‐29 colon cancer cell line viability was seen in a concentration‐dependent manner (the lowest cell viability of 32% at 5 mg/mL). Overall, investigating the application of protein‐based hydrolysates is one of the possible strategies that govern their applied intentions as preservatives and nutraceuticals in the food and pharmaceutical industries.

## Graphical abstract

The main objective of this study is to study antioxidant, antimicrobial, and in vitro cytotoxic activities of functional bioactive peptides obtained by enzymatic hydrolysis of CPP using different enzymes such as pancreatin, pepsin, and trypsin.

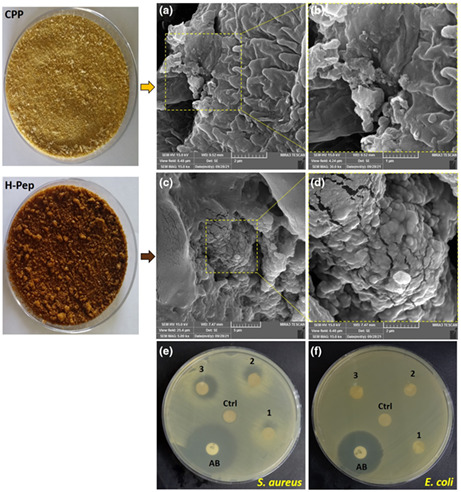

## 1 INTRODUCTION

Reactive oxygen species (ROS) such as OH radicals, singlet O, and H_2_O_2_ play important roles in modulating intracellular signal transduction pathways in biological systems. These ROS can be naturally blocked or eliminated by endogenous antioxidant defense systems in living organisms (Lorenzo et al., 2018). However, in some cases such as extreme environmental conditions, excessive ROS can be often generated, and if they are accumulated for a prolonged period, they will overwhelm the cellular natural antioxidant defense capacity resulting in oxidative damage to critical biological and vital macromolecules such as proteins, lipids, and DNA (Ravash et al., 2022). Likewise, accumulation of ROS free radicals in cells can lead to oxidative stresses resulting in disturbance of cell homeostasis and damage to the cell. Thus, an additional exogenous antioxidant protection system is necessary to maintain the oxidative balance status. Antioxidants, especially those supplied by dietary natural sources, are important to maintain redox homeostasis in cells of the human body. These natural‐based antioxidants show no undesirable adverse effects compared to chemically synthesized antioxidants (Nottagh et al., 2020; Wang et al., 2021).

Most of the peptides can offer antioxidant action similar to that of other well‐known antioxidants. Bioactive peptides obtained from plant protein sources have been well demonstrated to exhibit excellent antioxidant properties that could promote health‐promoting effects and prolong food shelf‐life beyond their basic nutritional value. The importance of characteristics of peptides derived from different food sources has been extensively discussed (Lorenzo et al., 2018). The outer layer of cereal grains contains secondary metabolites such as bioactive substances, which provide positive nutritional and health‐promoting properties in human body. Most of these bioactive compounds are concentrated in bran‐ or germ‐rich milling fractions, which can be separated, purified, and used as nutraceuticals. Different health‐promoting and functional properties including antioxidant, antihypertensive, and antitumor activities of bioactive peptides have been outlined in cereal protein hydrolysates (Peighambardoust et al., 2006, 2021). Proteins present in different sections of cereals are reported to contribute to biological functions such as antioxidant activities (Esfandi et al., 2019). Protein hydrolysates from cereal pollens also possess significant antioxidant and angiotensin‐converting enzyme (ACE) inhibitory activities (Maqsoudlou et al., 2019). Plant‐derived bioactive peptides are important functional ingredients that provide many health‐promoting effects, among which is preventing oxidative damages in living organisms (Akbarbaglu et al., 2021). Corn pollen is considered a nutritionally valuable resource, due to its rich sources of protein and fatty acids that are concentrated in the protective layer of pollen called “kitt zone” and this makes it play a significant role in preventing diseases such as hypertension, cancer, and diabetes (Chakrabarti et al., 2018). Health‐promoting and nutritional effects of corn pollen have led to its use as a natural additive in a wide range of foods (Albenzio et al., 2017). In this study, it is hypothesized that corn pollen is a rich source to produce bioactive peptides due to its different types of proteins.

Enzymatic hydrolysis is one of the most common methods used on a laboratory scale to produce bioactive compounds (Karami, Peighambardoust, Hesari, & Akbari‐Adergani, 2019). There are many studies reporting the hydrolysis of food proteins from different sources such as dairy, meat, egg, and plant byproducts proteins using trypsin (Deng et al., 2018), pepsin and papain (Xu et al., 2009), and alcalase (Maqsoudlou et al., 2019). Most of the studies confirm antioxidant and ACE inhibitory properties of functional peptides obtained from vegetable source proteins such as wheat germ proteins (Karami, Peighambardoust, Hesari, Akbari‐Adergani, & Andreu, 2019b). However, to the best of our knowledge, there is no study on properties of hydrolysates obtained from corn pollen protein (CPP) by different proteolytic enzymes yet. In this study, it is hypothesized that different proteases may affect functional properties of protein hydrolysates from CPP. Thus, the main objective of this study was to compare antioxidant and antibacterial potential of functional peptides obtained by enzymatic hydrolysis of CPP using different enzymes such as pancreatin, pepsin, and trypsin.

## 2 MATERIALS AND METHODS

### 2.1 Materials

The floral corn (*Zea mays* L. *saccharata*) pollen was gathered from maize farms in Gorgan (Golestan, Iran) in June 2020. Pollens were dehydrated at a temperature of below 40°C, then stored until used. Chemicals including ABTS (2,2′‐azino‐bis (3‐ethylbenzothiazoline‐6‐sulfonic acid)), Trolox (6‐hydroxy‐2,5,7,8‐tetramethychroman‐2‐carboxylic acid), DPPH (1,1 Diphenyl‐2‐picrylhydrazyl), TBA (thiobarbituric acid), ferrozine (3‐(2‐pyridyl)‐5‐6‐diphenyl‐1,2,4‐triazine‐4′,4″ disulphonic acid sodium salt), TCA (Trichloroacetic acid), pyrocatechol violet, and Coomassie brilliant blue (G250) were purchased from Sigma Aldrich Co (St. Louis, Missouri, USA). Hydrogen peroxide (H_2_O_2_), potassium persulfate (K_2_S_2_O_8_), ferrous sulfate (FeSo_4_), ferric chloride (FeCl_3_), ferrous chloride (FeCl_2_) copper sulfate (CuSo_4_), pyridine, dimethyl sulfoxide (DMSO), EDTA (ethylenediaminetetraacetic acid), and potassium ferricyanide provided by Merk (Darmstadt, Germany). Pepsin, trypsin, and pancreatin were obtained from Novo Nordisk A/S (Bagsværd, Denmark).

### 2.2 Corn pollen protein (CPP) extraction

Corn pollen was defatted using N‐hexane (at a ratio of 1:4 w/v) for 48 h at room temperature. The pollen solids were vacuum filtered from the suspension. Defatted pollen solids were then dispersed in distilled water (at 10% w/v) at pH = 10. Protein extraction was carried out for 1 h under magnetic stirring (250 rpm). The pH of the resulting protein solution decreased to 4.2 to precipitate proteins. The extracted CPP suspension was centrifuged (11,200 x *g*, 30 min), then lyophilized (Free Zone 4.5, Labconco, Kansas City, MO, USA) at −60°C for 72 h, and stored at −18°C inside zip‐lock bags until use.

### 2.3 Enzymatic hydrolysis

Enzymatic hydrolysis can be affected by a variety of factors, such as temperature, pH, and concentration. Enzymes work best within specific temperature and pH ranges, and suboptimal conditions can cause an enzyme to lose its ability to bind to a substrate. An increase in temperature generally speeds up enzymatic reactions. However, extreme high temperatures can cause an enzyme to denature. Moreover, every enzyme has an optimum pH range for best performance. Changing the pH outside of this range will slow enzyme activity. Increasing enzyme concentration will speed up the reaction, as long as there is substrate available to bind to. In this study, pancreatin, trypsin, and pepsin enzymes were used for enzymatic hydrolysis of corn pollen protein. The conditions of the hydrolysis process were selected based on the optimal activity point of each enzyme previously tested and optimized in our laboratory (Akbarbaglu et al., 2022).

#### 2.3.1 Pancreatin and trypsin

To perform enzymatic hydrolysis, defatted CPP powder (15.2% crude protein) was dissolved in 100 mL of 0.2 M potassium phosphate buffer (pH = 7.4) at a concentration of 10% (w/v) inside an Erlenmeyer flask. An amount of 0.2 g (2% w/v) pancreatin or trypsin in powder form was individually added and allowed to completely hydrate on a magnetic stirrer (250 rpm) keeping at a constant temperature of 37°C. The reaction was performed over a period of 120 min. At the end of the enzymatic hydrolysis process, the thermal process was performed in a water bath at 90°C for 20 min to inactivate peptidases. The samples were then cooled down to room temperature. The obtained suspension was then centrifuged at 5000 × *g* for 30 min and the supernatants were then lyophilized (Free Zone 4.5, Labconco, Kansas City, MO, USA) at −60°C for 72 h, and stored at −18°C inside zip‐lock bags until use.

#### 2.3.2 Pepsin

To perform pepsin hydrolysis, the same procedure was used as explained in Section 2.3.1, except that 0.1 M acetate buffer (pH = 3) was used to provide optimal conditions for enzymatic hydrolysis.

### 2.4 Determining the degree of hydrolysis

To measure DH, CPP hydrolysate suspension and trichloroacetic acid (TCA, 0.45 M) were mixed at a ratio of 1:1 (v/v). The mixture was then incubated at 4°C for 10 min and centrifuged (5000 × *g*) for 15 min. The soluble proteins were estimated using Coomassie brilliant blue dye‐binding methods, according to the standard curve plotted based on different concentrations of bovine serum albumin. The degree of hydrolysis (DH) was calculated based on the following equation:

(1) $$\mathrm{DH}\;\left(\%\right)=\frac{\text{Protein}\;\left(\mathrm{TCA}+\text{Supernatant}\right)}{\text{Protein}\;\left(\text{corn pollen hydrolysate suspension}\right)}\times 100$$

### 2.5 FT‐IR spectroscopy

FT‐IR spectroscopy was used to assess possible changes in chemical structure of native pollen protein compared to its hydrolysates as action of different enzymes (Peighambardoust et al., 2007). For this purpose, protein or hydrolysates powders were mixed with KBr (1:100 w/w) and compressed to make pellet disks. Infrared spectrum was recoded using spectrophotometer (Bruker‐Tensor 27, Bremen, Germany) covering wavenumber range 4000–400 cm^−1^ at 4 cm^−1^ resolution (Soltanzadeh et al., 2021a, 2021b).

### 2.6 Amino acid composition of CPP and hydrolysates

The effect of proteolytic action of different proteases on amino acid composition of CPP and its hydrolysate was studied by reversed‐phase (RP) HPLC (Young Lin Acme 9000, YL Instruments, Anyang, Korea) equipped with an RP column (150 mm × 4.6 mm × 0.5 mm, Teknokroma, RP‐C18 ODS‐A, Barcelona, Spain) and a fluorescence detector (LC305. Lab Alliance. State College, PA, USA) (Peighambardoust et al., 2008; van der Goot et al., 2008). Acetate buffer was run as mobile phase with a flow rate of 1.3 mL min^−1^. First, the hydrolyzed samples were diluted with solution A (125 mM borate, pH = 9.4) at a ratio of 1:20 followed by adding methanol and L‐homoserine. The present mixture was blended with solution B (O‐Phthaldialdehyde + Borate + Mercaptoethanol + MeOH) and C (0.75 M HCl) and was injected into the RP‐HPLC with a Hamilton syringe. Total amino acid residues were determined after hydrolysis with 6 N HCl at 110°C for 24 h. CPP samples previously hydrolyzed by the enzymes did not undergo the hydrolysis process and the amino acid composition was expressed as mg g^−1^ dry sample (Ngamsuk et al., 2020).

Amino acid composition can be used as an indication of nutritional and functional groups of AA in CPP hydrolysates compared to its native protein according to Equations (2–5):

(2) $$\text{Essential amino acids}\;\left(\mathrm{EAA}\right)=\mathrm{Thr}+\mathrm{Met}+\mathrm{Val}+\mathrm{Leu}+\mathrm{Ile}+\mathrm{Trp}$$

(3) $$\text{Antioxidant amino acids}\;\left(\mathrm{AAA}\right)=\mathrm{Trp}+\mathrm{Met}+\mathrm{His}+\mathrm{Tyr}+\mathrm{Lys}$$

(4) $$\text{Hydrophilic amino acids}\;\left(\text{HyPhi}-\mathrm{AA}\right)=\mathrm{Asp}+\mathrm{Glu}+\mathrm{Arg}+\mathrm{His}+\mathrm{Ser}+\mathrm{Thr}$$

(5) $$\text{Hydrophobic amino acids}\;\left(\text{HyPho}-\mathrm{AA}\right)=\mathrm{Ala}+\mathrm{Val}+\mathrm{Ile}+\mathrm{Leu}+\mathrm{Tyr}+\mathrm{Phe}+\mathrm{Trp}+\mathrm{Met}$$

### 2.7 SDS‐PAGE (sodium dodecyl sulfate–polyacrylamide gel electrophoresis)

Variation in molecular weight (Mw) of the chemical components of CPP hydrolysates was characterized by SDS‐PAGE according to the method described by Mechmeche et al. (2017) with alteration as explained below. Polyacrylamide (PA) gel with a concentration of 5 and 12.5% was used for stacking and running gels, respectively. Dispersions of hydrolysates in DW (0.2% w/v) and a buffer containing bromophenol blue at a ratio of 1:1 were prepared, which was then heated at 90°C for 10 min followed by cooling to ambient temperature. Aliquots of 20 μL of sample and 5 μL of a protein marker (Mw of 20–120 kDa) were injected into electrophoresis wells. The gel was then placed in the set‐up. SDS‐PAGE was carried out at a constant voltage of 70 and 120 V for stacking and running gels, respectively. When the reagent line reached the bottom of the gel, the process was ended and the gel was then removed and stained by Coomassie brilliant blue (R‐250) (10% w/v) overnight to observe the protein bands.

### 2.8 Evaluation of the antioxidant activity (AA) of hydrolysates

#### 2.8.1 DPPH free radical scavenging

To evaluate DPPH free radical scavenging activity (RSA), 1.5 mL hydrolysate solution at a concentration of 10 mg mL^−1^ was blended with 1.5 mL of 0.2 mM alcoholic solution of DPPH. The obtained solution was then stored in dark for 40 min. The mixture was then centrifuged (5000 × g, 15 min) and the supernatant was taken and the adsorption was measured by a UV–vis spectrophotometer (Varian Cary 500, Agilent Technologies, Santa Clara, CA, USA) at 517 nm. DPPH free radical scavenging was estimated from the following equation (Fasihnia et al., 2020; Shahi et al., 2020).

(6) $$\text{Inhibition}\;\left(\%\right)=\left[1-\frac{A_{\text{sample}}}{A_{\text{blank}}}\right]\times 100$$

where *A*_sample_ and *A*_blank_ are the absorbance (at 517 nm) of hydrolysate and the control, respectively.

#### 2.8.2 ABTS ^+^ free radical scavenging and Trolox equivalent antioxidant capacity (TEAC)

To evaluate ABTS^+^ radical scavenging activity (RSA), potassium persulfate (2.45 mM) and ABTS^+^ (7.45 mM) solutions were combined and kept at dark for 16 h. the resulting solution was then diluted with 0.2 M PBS to an absorbance of 0.70 at 734 nm. Then, 30 μL of hydrolysate solution was mixed with 3 mL of ABTS^+^ solution, shortly vortexed (30 s), and left in dark for 6 min (Mohammadi et al., 2022). ABTS^+^ radical scavenging activity was measured using Equation (6), except that the absorbance was read at 734 nm. To determine the Trolox equivalent antioxidant capacity (TEAC), different concentrations of Trolox (50–1000 μM) were prepared and a standard curve of the reaction with ABTS was plotted (Fasihnia et al., 2018; Nottagh et al., 2018).

#### 2.8.3 Hydroxyl radical scavenging activity

Evaluation of hydroxyl radical scavenging activity was performed using α‐deoxyribose oxidation. For this purpose, 0.2 mL of 10 mM Fe_2_SO_4_‐EDTA solution was added to 0.5 mL of 10 mM α‐deoxyribose. Then, hydrolysate sample (0.2 mL), sodium phosphate buffer (0.2 M, 0.9 mL), and hydrogen peroxide (10 mM, 0.2 mL) were mixed in a reaction mixture and incubated at 37°C for 1 h. An aliquot of 1 mL trichloroacetic acid (2.8% w/v) was added to the mixture. Then, 1 mL of thiobarbituric acid (1% w/v) was added to stop the reaction. The resulting mixture was heated to boiling for 15 min followed by cooling to ambient temperature. The mixture was centrifuged (5000 × *g* for 10 min) and the absorption of the supernatant was recorded at 532. In the blank sample, the equivalent volume of water was used. The hydroxyl radical inhibition was calculated using Equation (6) at absorbance of 532 nm.

#### 2.8.4 Reducing power activity assay

To evaluate antioxidant activity of peptides, the reduction of Fe^+3^/ferricyanide complex to the ferrous form can be used as reducing power activity. For this purpose, hydrolysate sample (0.5 mL), phosphate buffer (0.5 mL, pH = 6.6), and potassium ferricyanide (0.5 mL at 10 mg mL^−1^) were mixed and incubated for 20 min at 50°C. Then, 0.5 mL of 10% trichloroacetic acid (10% w/v) was added and the resulting mixture was centrifuged (5000 × *g*) for 10 min. The obtained supernatant was diluted with 1 mL distilled water and 0.2 mL ferric chloride (0.1% w/v) and kept at room temperature for 10 min and its absorption was obtained at 700 nm. As a blank sample, the equivalent volume of water was used instead of the original sample (Shahidi & Zhong, 2015). The reducing power activity was calculated using Equation (6) at absorbance of 700 nm.

#### 2.8.5 Fe^2+^ chelating activity

To measure Fe^2+^ chelating activity of protein hydrolysates, the sample (1 mL), iron (ІІ) chloride solution (0.05 mL at 2 mM), double‐distilled water (1.85 mL), and ferrosine (0.1 mL at 5 mM) were combined and stirred vigorously at room temperature for 10 min. The absorbance of the resulting mixture was read at 562 nm (Shahi et al., 2020). The Fe^2+^ chelating activity was calculated from Equation (6) considering 562 nm absorption wavelength.

#### 2.8.6 Cu^2+^ chelating activity

To determine Cu^2+^ chelating activity of hydrolyzed CPP, hydrolysate sample (1 mL) and CuSo_4_ solution (1 mL at 0.2 mM) were mixed and incubated for 5 min at room temperature. Then, 1 mL of 10% acetic acid solution was added to the reaction mixture. The sample was then centrifuged (5000 × *g*) for 10 min and the supernatant (0.2 mL) was transferred to the microtube tube (5 mL) followed by adding 1 mL of 10% pyridine and 20 μL of 0.1% pyrocatechol. The mixture was then incubated for 5 min and the adsorption was read at 632 nm. The Cu^2+^ chelating activity was calculated using Equation (6), considering absorbance at 632 nm (Sarabandi et al., 2017).

#### 2.8.7 Total antioxidant activity (TAA)

This method is based on the reduction of molybdenum (VI) to molybdenum (V). It is a capacity that is associated with the formation of the green complex of phosphomolybdenum in acidic pH. To measure TAA, a mixture containing 0.1 mL sample solution (10–50 mg mL^−1^) and 1 mL of reagent (0.6 M sulfuric acid, 28 mM sodium phosphate, and 4 mM ammonium molybdate) was prepared and incubated in a water bath set to 90°C for 90 min. After cooling to ambient temperature, the absorbance was measured at 695 nm. Double distilled water was used as a control (Sarabandi et al., 2018). TAA activity was calculated using Equation (6) considering absorbance at 695 nm (Soltanzadeh et al., 2022).

### 2.9 CPP and hydrolysates morphology

The Morphology of CPP and the hydrolyzed samples were monitored by scanning electron microscopy (SEM) (MIRA3, TESCAN, Brno, Czech Republic). A very small amount of powder samples was attached to an aluminum plate using double‐sided adhesive tape. It was then covered with a thin layer (10 nm) of gold using a vacuum ion sputtering device (E−1010, Hitachi, Japan). Finally, the coated samples were observed under a microscope at an accelerating voltage of 15 kV.

### 2.10 Antibacterial effect

A disk diffusion method was used to investigate the antibacterial properties of CPP and hydrolyzed samples against *Escherichia coli* (ATCC 25922) and *Staphylococcus aureus* (ATCC 25923) (Golshan Tafti, Peighambardoust, Behnam, et al., 2013; Golshan Tafti, Peighambardoust, Hesari, et al., 2013). First, a concentration of half McFarland of the bacteria was transferred to culture plates. The primary and hydrolyzed protein of corn pollen was transferred to the culture medium by pepsin enzyme at concentrations of 50, 100, and 200 mg mL^−1^. The antibiotic ciprofloxacin was used as a positive control and a blank disk was used as a negative control. Finally, the plates were incubated at 37°C for 24 h. Antibacterial effect of samples was expressed as the diameter of inhibition zone (DIZ) in mm (Khodaeimehr et al., 2018).

### 2.11 Cytotoxic and antiproliferative activity

The MTT (3‐(4,5‐dimethylthiazole‐2‐yl)‐2,5‐diphenyltetrazolium bromide) is a colorimetric method for assessing cell metabolic activity against bioactive compounds. In this study, MTT test was carried out to evaluate the effect of CPP hydrolysates proteolyzed by pepsin enzyme on colon cancer cells (HT‐29 line). MTT determines the amount of cell activity by reducing the yellow color and the formation of insoluble formazan crystals. Cytotoxic activity of hydrolysates was carried out as described by Vaucher et al. (2010) with slight alterations explained below. Briefly, cancer cells (HT‐29 at 2.5 × 10^5^cells mL^−1^) were seeded in a 96‐well plate and exposed to different serial concentrations of H‐Pep and CPP samples (0.625–5 mg mL^−1^). SDS as positive control (at 20 mg mL^−1^) was also added to wells. Fresh minimum essential medium (MEM) (Sigma‐Aldrich, St. Louis, Missouri, USA) was used as negative control. Plates were incubated for 24 h at 37°C. Then, the medium was drawn out from all wells and aliquots of 50 μL MTT solution (2 mg mL^−1^) prepared in MEM were added to wells, and the plates were incubated for 4 h at 37°C. The MTT solution was then removed with extra care without disturbing the cell contents, and 50 μL of DMSO was added to each well to dissolve the crystals of formazan. To complete the crystal dissolution, the plates were gently shaken for 5 min. The absorbance was read on a microplate absorbance reader (Bio‐Rad Laboratories, Hercules, CA, USA) at 540 nm. The viability of cells was obtained from the following Eq.:

(7) $$\text{Viability}\;\left(\%\right)=\left[\frac{A_{\mathrm{TS}}}{A_{B}}\right]\times 100$$

where A_TS_ and A_B_ are the absorbances of treated samples and blank, respectively.

### 2.12 Statistical analysis

In this study, statistical data were analyzed using Statistical Analysis System ver. 9.2 (SAS Inc., Cary, NC, USA). A one‐way analysis of variance (ANOVA) and Tukey's multiple‐range test at a significance level of *p* < .05 were used. Triplicate measurements were performed for all analysis.

## 3 RESULTS AND DISCUSSION

### 3.1 Degree of hydrolysis (DH)

**FIGURE 1:**
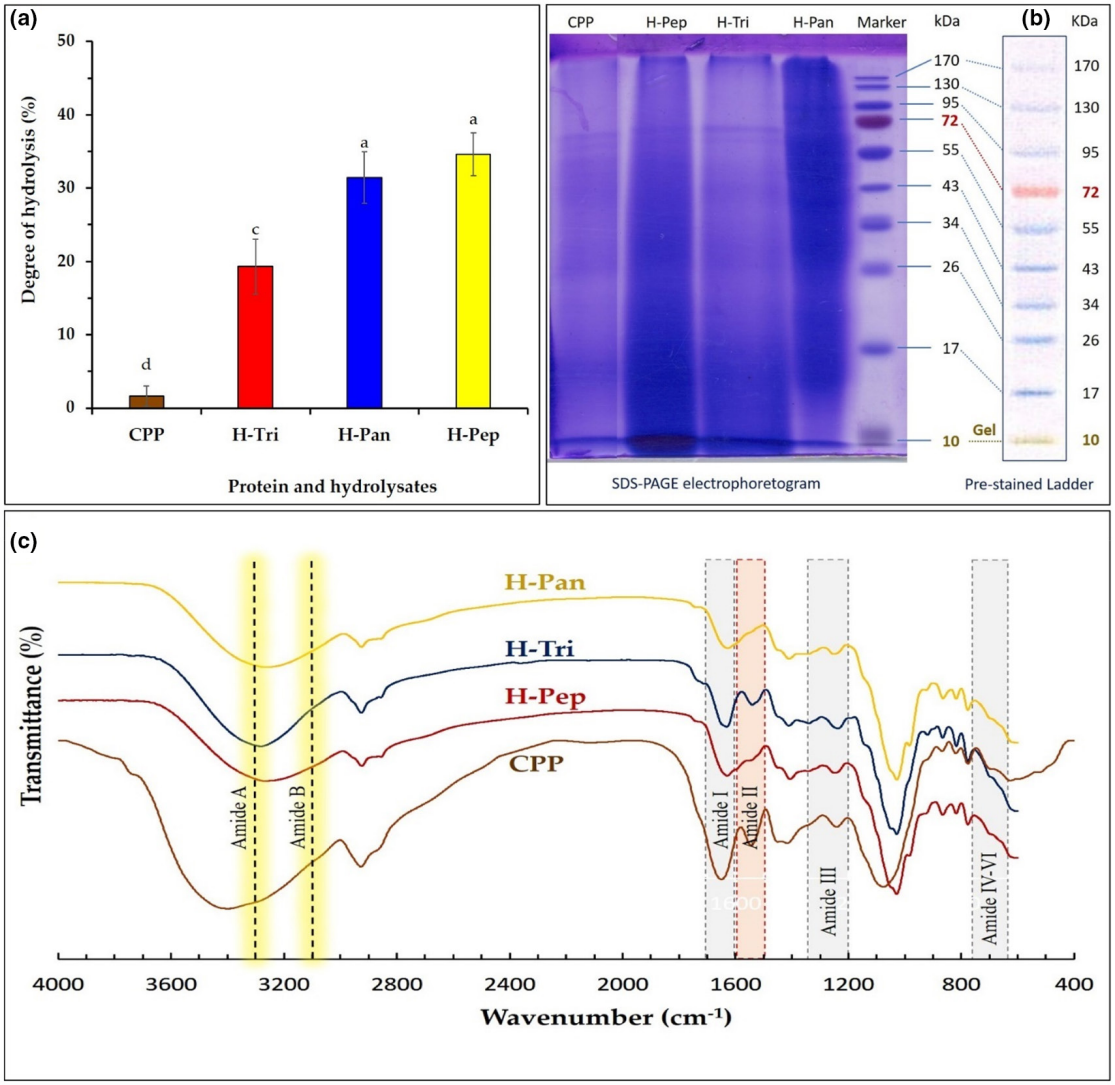
(a) The degree of hydrolysis of corn pollen protein (CPP) and its hydrolysates obtained by pepsin (H‐Pep), trypsin (H‐Tri), and pancreatin (H‐Pan) under conditions described in Section 2.3. Data are the mean of triplicate measurements. Error bars represent standard deviations. Different alphabetical letters show significant (*p* < .05) differences between means. (b) SDS‐PAGE results of CPP before and after enzymatic hydrolysis by pancreatin (H‐Pan), pepsin (H‐Pep), and trypsin (H‐Tri). Marker protein bands measured by electrophoresis setup matching with the Mw of prestained PAGE ruler have been shown for the matter of comparison. (c) FT‐IR spectra of primary corn pollen protein (CPP) and its hydrolysates obtained by the action of pepsin (H‐Pep), trypsin (H‐Tri), and pancreatin (H‐Pan).

Degree of hydrolysis is a direct indication of hydrolysis extent and chain length (molecular weight) of protein hydrolysates. It can, thus, determine technofunctional and biological properties of the obtained peptides. Higher DH values correspond to peptides with shorter chain lengths. DH is greatly affected by enzyme type and proteolysis conditions. Figure 1a shows variation in DH as a function of different enzymes. At the proteolysis conditions used in this study, pepsin and pancreatin enzymes showed significantly (*p* < .05) higher DH values compared to that of trypsin. The protein hydrolysis efficiency of about 65%, 64%, and 60% were obtained for pepsin, pancreatin, and trypsin, respectively. There was no significant (*p* > 0.05) difference between proteolytic action of pancreatin and pepsin. Primary CPP showed no proteolysis (with the lowest DH value). Differences in proteolytic action of different enzymes may be related to the exo‐ and endoproteinase activities of the enzymes (Malomo et al., 2015).

### 3.2 SDS‐PAGE

SDS‐PAGE results of CPP before and after enzymatic hydrolysis by pancreatin, pepsin, and trypsin are shown in Figure 1b. The figure demonstrates a molecular weight range 10–72 kDa for all samples. Unhydrolyzed CPP exhibited several major bands mostly located at Mw of 72, 55, and 26 kDa, whereas hydrolysates demonstrated different electrophoretic patterns compared to their primary CPP. For example, in H‐Pep sample, most of high Mw bands of primary CPP disappeared indicating a major breakdown of initial protein structure to low Mw peptides and amino acids. In H‐Pan hydrolysate, 130 and 95 kDa bands of primary CPP have been lost indicating protein breakdown. These results are in good agreement with DH results presented in Figure 1A, confirming a hydrolysis extent order of H‐Pep > H‐Pan > H‐Tri.

It can be shown by DH and SDS‐PAGE analysis that the hydrolyzed peptides are generated from corn pollen hydrolysis reaction. Bioactive peptides are fragments (composed of 2–20 amino acids) buried in the amino acid sequence of the primary proteins. However, these parts are inactive in the primary protein structure. To release and activate these peptides, hydrolysis is performed using chemical or enzymatic methods. The release of these parts leads to their functional and antioxidant properties. However, the characteristics of the produced peptides are affected by the different performances of each enzyme (Bhandari et al., 2020; Lorenzo et al., 2018).

SDS‐PAGE analysis used in other studies showed proteins' breakdown to low‐molecular‐weight peptides for ovomucin (Abeyrathne et al., 2016), sodium caseinate (Luo et al., 2014), black cumin (Shahi et al., 2020), and faba bean proteins (Samaei et al., 2020) by proteolytic action of pepsin, trypsin, alcalase, and pancreatin enzymes.

### 3.3 FTIR spectroscopy

The chemical structure of a biological compound can be determined using FT‐IR spectroscopy based on IR radiation wavelength and intensity absorbed by the sample. This technique is widely applied for characterization of the secondary structure of proteins or polypeptide and their hydrolysates. Effect of the activity of different enzymes on structural changes of CPP and its hydrolysates was investigated using FT‐IR spectroscopy (van der Goot et al., 2008).

Figure 1c illustrates FT‐IR spectra of a CPP and its hydrolysates. Six characteristic IR bands of amides A, B, and I−VI were characterized. The amide I and II bands are the two most prominent vibrational bands of the protein backbone. Of these two, amide I is regarded as the most sensitive spectral region (1700–1600 cm^−1^) in the secondary structural components of polypeptides, mostly attributing to the C=O stretching vibrations of the peptide linkages. Amide І band found in primary protein (CPP) at 1660 cm^−1^ was slightly shifted to 1630 cm^−1^ in hydrolyzed samples (H‐Pep, H‐Tri, and H‐Pan), indicating that C=O groups of peptide linkages have not been greatly affected by hydrolysis conditions used in this study. The amide II band region (1600–1500 cm^−1^) is mainly attributed to in‐plane NH bending and the CN stretching vibration, exhibiting less protein conformational sensitivity than amide I counterpart (Kong & Yu, 2007). As can be seen from Figure 1c, the most noticeable changes in IR spectra of hydrolysate samples have occurred in amide II region. A distinct band of amide II in CPP sample (at 1550 cm^−1^) has disappeared in H‐Pep and H‐Pan samples, indicating conformational changes in the protein structure as affected by the action of pepsin and pancreatin enzymes. Trypsin had apparently little effect on the amide II region‐related changes. Similar results were reported for changes in the secondary structure of *Spirulina platensis* protein (in amid I and II regions) affected by the action of pepsin and pancreatin (Akbarbaglu et al., 2022).

The amide III band (1210–1320 cm^−1^) is attributed to alpha‐helix, beta‐sheet, beta‐turn, and random coils structures (Akbarbaglu et al., 2022). The intensity of this band in H‐Pep and H‐Pan samples was lower than that of obtained for primary CPP. Similar to amide II region, trypsin enzyme had minimal effect on IR absorbance related to amide III region (Figure 1c). Other amide bands (amides A, B, and IV–VI) are reported to attribute to the nature of side chains and hydrogen bonding, which are less important in the protein conformational structure than C=O stretching and NH bending vibrations (Kong & Yu, 2007). These amide bands have not been affected by the action of different enzymes under proteolysis conditions used in this study. In general, structural changes in FT‐IR spectra can be related to hydrolysis conditions such as temperature and pH. Storage conditions, amino acid content, and hydrolysis temperature are also other influencing factors that change the secondary structures of proteins such as α‐helix, β‐sheet, and β‐turn.

### 3.4 Amino acid composition

**FIGURE 2:**
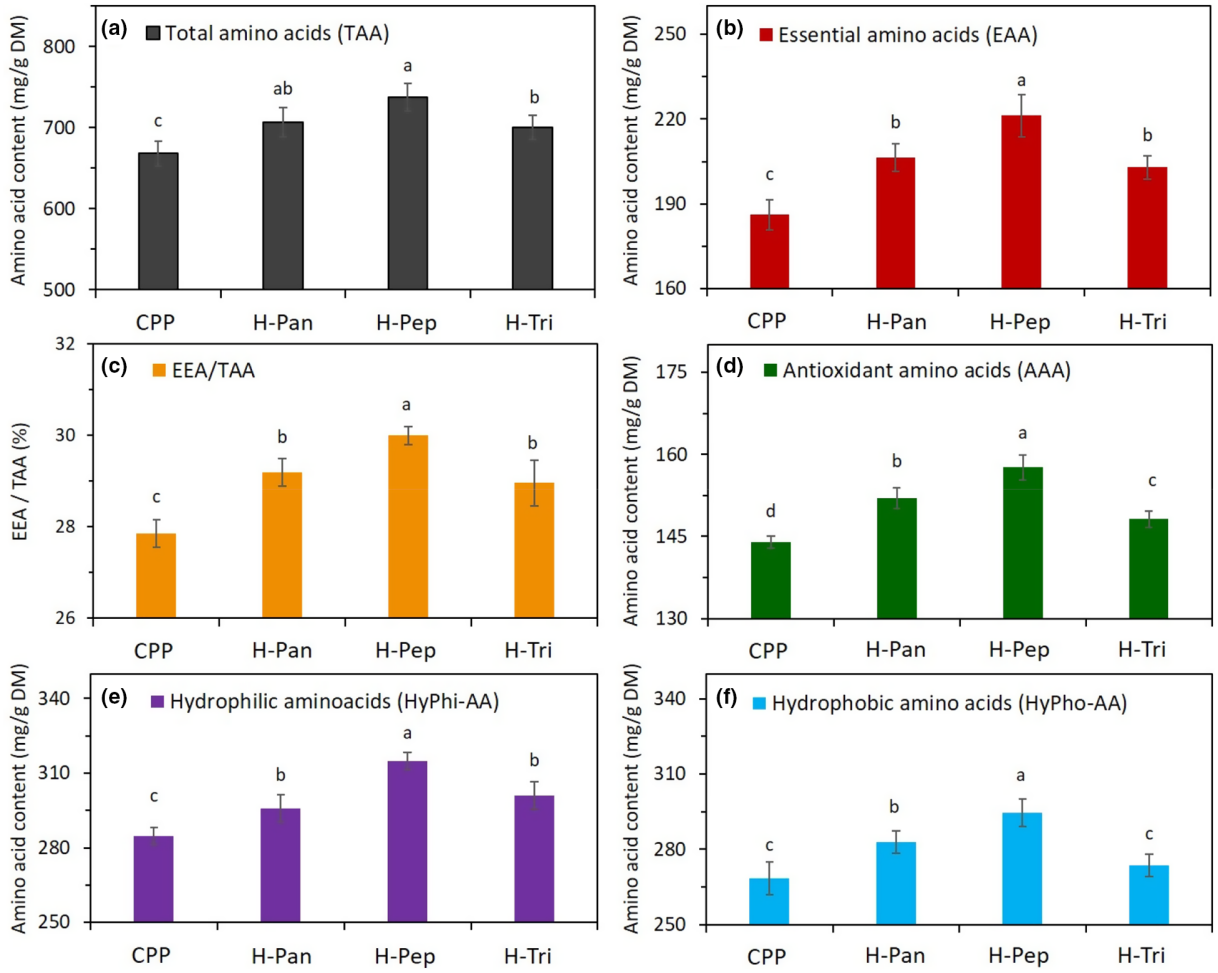
Amino acid composition of corn pollen protein (CPP) and its hydrolysates obtained by pepsin (H‐Pep), trypsin (H‐Tri), and pancreatin (H‐Pan) under conditions described in Section 2.3. Data are the mean of triplicate measurements. Error bars represent standard deviations. Different alphabetical letters show significant (*p* < .05) differences between means.

**FIGURE 3:**
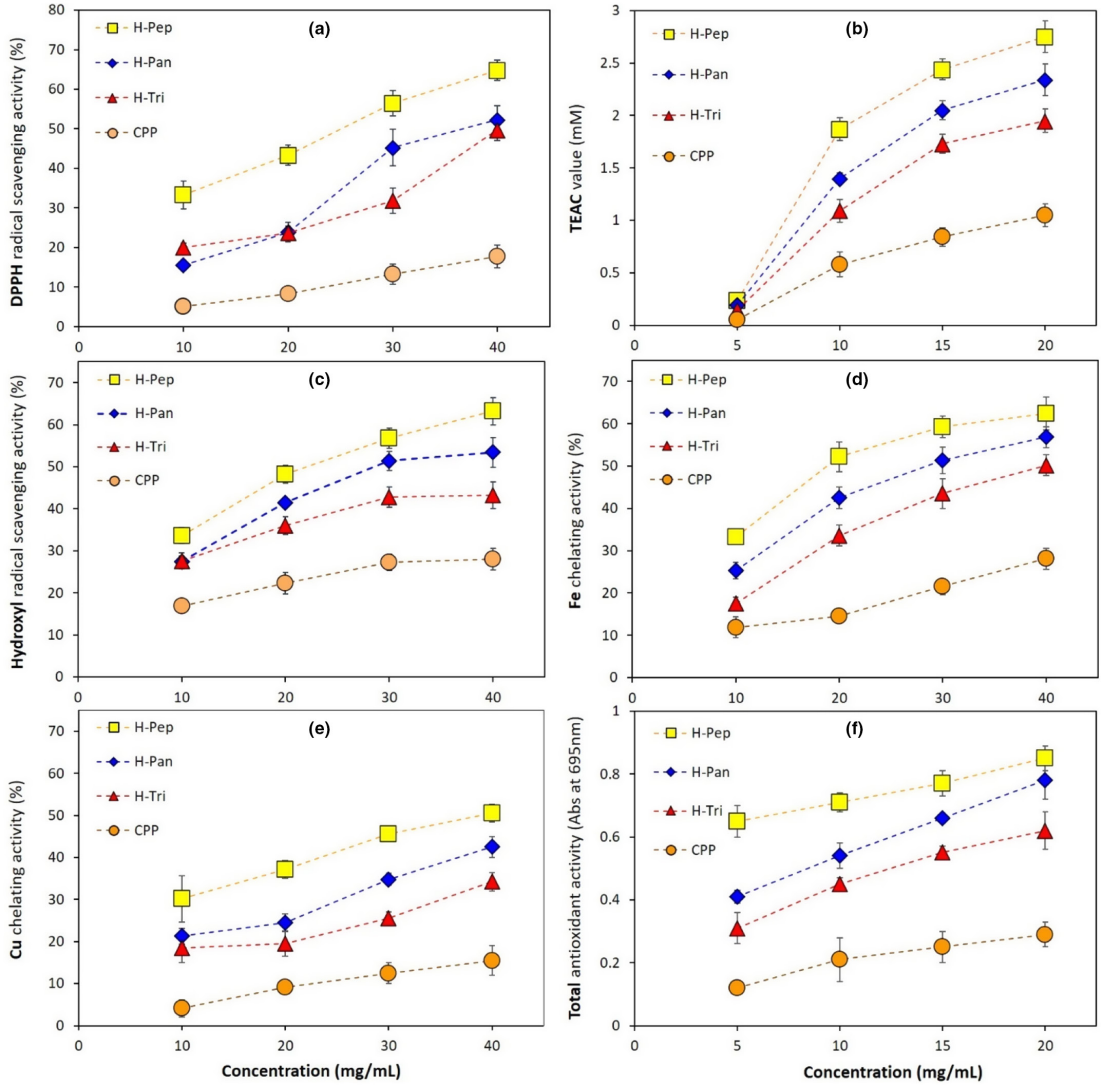
Effect of hydrolysate types obtained by the action of pepsin (H‐Pep), trypsin (H‐Tri), and pancreatin (H‐Pan) and their concentration on (a) DPPH radical scavenging activity, (b) Trolox equivalent antioxidant capacity, (c) hydroxyl radical scavenging, (d) Fe chelating activity, (e) Cu chelating activity, and (f) total antioxidant activity. Data are the mean of triplicate measurements. Error bars represent standard deviations.

The amino acid composition (AAC) of proteins or polypeptides plays an important role in their both nutritional and technofunctional properties. Table 1 provides detailed information about different types of amino acids, for example, essential (Thr + Met + Val + Leu + Ile + Trp), antioxidant (Trp + Met + His + Tyr + Lys), hydrophobic (Asp + Glu + Arg + His + Ser + Thr), and hydrophilic (Ala + Val + Ile + Leu + Tyr + Phe + Trp + Met) amino acids of primary CPP and its hydrolysates obtained by the proteolytic action of pepsin, trypsin, and pancreatin enzymes. The content of these groups of functional amino acids for different hydrolysates and their primary protein was calculated from this table and has been compared in Figure 2. As can be seen in this figure, essential (EAA) and antioxidant amino acids (AAA) were significantly (*p* < .05) higher in all hydrolysates than those of CPP. Among hydrolysate samples, H‐Pep showed significantly (*p* < .05) higher contents of AAA, EAA, and charged (hydrophobic and hydrophilic) amino acids compared to the other two types of hydrolysates. This can be due to different proteolytic actions of enzymes applied in this study. Our results were in line with those by Akbarbaglu et al. (2022) who reported that hydrolysate samples obtained from *Spirulina platensis* protein by the action of pepsin and pancreatin had higher contents of functional amino acids (EAA, AAA, and charged AA). Due to chemical structure and functional groups of these amino acids, they are capable of scavenging free radicals and can act as an effective antioxidant in biological cells. Thus, hydrolysates samples, especially those produced by pepsin and pancreatin, are expected to show effective in vitro antioxidant activity against free radicals. As can be seen in Figure 3c, the ratio of EAA to TAA in H‐Pep and H‐Pan hydrolysates was higher than those of other samples. This value of hydrolysate samples accounts for almost 30%, which is close to that recommended by FAO/WHO (32%).

**TABLE 1:** Amino acid composition (mg g^−1^ DM) of corn pollen protein (CPP) and its hydrolysates obtained by pancreatin (H‐Pan), pepsin (H‐Pep) and trypsin (H‐Tri).

Amino acid type	CPP	H‐Pan	H‐Pep	H‐Tri
Aspartic acid (Asp)^H+^	91.2 ± 3.6*	91.6 ± 3.1	95.6 ± 1.6	92.3 ± 1.2
Glutamic acid (Glu)^H+^	100.3 ± 2.4	103.1 ± 1.8	110.4 ± 3.0	107.3 ± 2.6
Asparagine (Asn)	‐	7.6 ± 0.6	6.3 ± 0.8	6.2 ± 0.5
Histidine (His)^A,H+^	23.8 ± 0.3	24.7 ± 0.5	25.2 ± 0.3	26.4 ± 0.2
Serine (Ser)^H+^	7.1 ± 0.1	7.5 ± 0.0	8.9 ± 0.0	7.4 ± 0.1
Glutamine (Gln)	‐	7.6 ± 0.0	9.3 ± 0.0	6.8 ± 0.0
Arginine (Arg)^H+^	31.6 ± 0.5	32.7 ± 0.7	33.8 ± 0.5	33.5 ± 0.4
Citrulline (Cit)	‐	‐	‐	3.5 ± 0.1
Glycine (Gly)	43.2 ± 2.6	35.1 ± 3.1	35.7 ± 2.5	34.1 ± 1.9
Threonine (Thr)^E,H+^	30.6 ± 0.6	36.2 ± 1.0	40.9 ± 1.2	34.2 ± 0.4
Alanine (Ala)^H‐^	48.3 ± 1.3	43.3 ± 1.1	42.7 ± 1.5	40.4 ± 1.7
Tyrosine (Tyr)^A,H‐^	30.4 ± 0.8	33.2 ± 1.1	34.4 ± 1.0	29.1 ± 0.7
Methionine (Met)^E,A,H‐^	12.8 ± 0.3	13.9 ± 0.2	15.7 ± 0.4	12.7 ± 0.4
Valine (Val)^E,H‐^	45.3 ± 1.2	48.6 ± 1.1	50.9 ± 1.7	46.4 ± 1.3
Phenylalanine (Phe)^H‐^	34.2 ± 1.0	36.2 ± 0.9	37.2 ± 0.5	35.3 ± 0.4
Isoleucine (Ile)^E,H‐^	32.1 ± 1.1	38.7 ± 0.4	40.1 ± 1.0	38.3 ± 0.7
Leucine (Leu)^E,H‐^	60.1 ± 2.0	62.1 ± 2.1	65.4 ± 1.8	63.2 ± 1.0
Ornithine (Orn)	‐	4.3 ± 0.2	2.5 ± 0.1	3.3 ± 0.2
Lysine (Lys)^A^	71.8 ± 1.9	73.4 ± 1.1	74.1 ± 1.0	71.9 ± 1.5
Tryptophane (Trp)^E,A,H‐^	5.2 ± 0.1	6.8 ± 0.1	8.2 ± 0.3	8.1 ± 0.3
Total amino acids (TAA)	668.0 ± 19.0	706.6 ± 17.9	737.3 ± 17.0	700.4 ± 15.1

* Data are mean ± SD values; ^E^Essential amino acids (EAA); ^A^Antioxidant amino acids (AAA); ^H+^Hydrophilic amino acids (HyPhi‐AA); ^H−^Hydrophobic amino acids (HyPho‐AA).

### 3.5 Antioxidant activity

#### 3.5.1 DPPH and ABTS ^+^ radical scavenging activity

The effect of enzyme type and different peptide concentrations on antioxidant activity in terms of DPPH (anionic and lipophilic radicals, Figure 3a) and ABTS (cationic and hydrophilic radicals) based on the TEAC value was studied. It is clear from these figures that all hydrolysates showed significantly (*p* < .05) higher free radical scavenging activity compared to primary CPP. Among hydrolysates, H‐Pep had the most enhanced antioxidant activity followed by H‐Pan and H‐Tri samples. Moreover, the antioxidant activity was significantly affected by the concentration of peptides (bioactive compounds in hydrolyzed samples) as increasing the concentration from 5 to 20 mg/mL led to an increase in the TEAC values (Figure 3b). Results in Figure 1a already showed that H‐Pep hydrolysates had higher DH values compared to other hydrolysates, indicating that more hydrophobic amino acid residues in these peptides are exposed, resulting in more available active sites to scavenge DPPH and ABTS radicals. These results can be also supported by the analysis of amino acid composition shown in Figure 2d, where H‐Pep samples contained the highest concentration of antioxidant amino acids (Trp, Met, His, Tyr, and Lys). These amino acids convert free radicals to their stable forms by proton or electron donation (You et al., 2009). Hydrolysates obtained from the action of pepsin and pancreatin in other sources of proteins, for example, mung bean (Xie et al., 2019), walnuts (Moghadam et al., 2020), and *Spirulina platensis* (Akbarbaglu et al., 2022; Mohammadi et al., 2022) showed enhanced antioxidant activity too.

#### 3.5.2 Hydroxyl radical scavenging

Hydroxyl radicals are important chemicals with the ability to adversely affect biological molecules such as DNA. The effect of hydrolysate type and concentration on OH radicals scavenging is shown in Figure 3c. The highest OH radical inhibition activity belonged to H‐Pep followed by H‐Pan and H‐Tri hydrolysates, particularly at their higher concentrations. These results are parallel to those obtained for DPPH and ABTS^+^ radical scavenging activity tests. As explained before, hydrolysates with lower Mw (higher DH) could expose more hydrophobic residues in the peptides, providing more reactive sites to scavenge free radicals, including OH radicals. Furthermore, antioxidant amino acids (Trp, Met, His, Tyr, and Lys), which are dominant in H‐Pep hydrolysates compared to other types (Figure 2d), could effectively scavenge free radicals. It is reported that certain amino acids, such as tryptophan containing an indole ring, act as a proton donor and could inhibit hydroxyl radicals (Shahidi & Zhong, 2015). Similar results were obtained by Cui et al. (2022), who reported that hydroxyl radicals were strongly scavenged by hydrolysates obtained from the enzymatic hydrolysis of milk proteins.

#### 3.5.3 Fe^2+^ and Cu^2+^ chelating activity

Metal ions are the accelerators of lipid peroxidation that can lead to food spoilage. Antioxidant peptides are among the compounds with antioxidant action providing effective Fe^2+^/Cu^2+^ chelating activity and lipid peroxidation inhibition (López‐García et al., 2022). Fe^2+^ and Cu^2+^ chelating activities of hydrolysates obtained by different enzymes are shown in Figure 3d,e, respectively. These results suggest that the type and concentration of peptides are important in providing Fe^2+^/Cu^2+^ chelating activity. The highest chelating activity of Fe^2+^ (62.5%) and Cu^2+^ (50.5%) was obtained in H‐Pep hydrolysate, followed by H‐Pan and He‐Tri types. These results followed the same trend obtained in previous sections for DPPH, ABTS, and OH radical scavenging activity (Figure 3a–c). The metal‐ions chelating activity of a peptide molecule can be influenced by various factors. The existence of acidic amino acids (aspartic and glutamic acid) as well as alkaline types (arginine and lysine) in a polypeptide provides more access to carboxylic and amino groups that results in an increased chelating activity of the peptides (Akbarbaglu et al., 2022).

#### 3.5.4 Total antioxidant activity (TAA)

The basis of this experiment is the reduction of molybdenum VI to molybdenum V under acidic conditions caused by sulfuric acid. The results (Figure 3f) showed that increasing the concentration of hydrolysates led to a significant increase in TAA values. Moreover, hydrolysates obtained from pepsin and pancreatin at a concentration of 20 mg mL^−1^ exhibited the highest antioxidant properties. At this concentration, H‐Pep, H‐Pan, and H‐Tri samples showed TAA values of 0.85, 0.78, and 0.62, respectively. These results followed the same trend obtained in previous sections for DPPH, ABTS, OH, and metal‐ions radical scavenging activity (Figure 3a–e). Similar results were obtained in other studies. For example, the total antioxidant activity of common bean proteins was significantly improved by enzymatic hydrolysis using a blend of proteases (Ohara et al., 2021).

In bee products and particularly in pollen, carbohydrates and their biological activities can be also considered. Polysaccharide‐based compounds remaining in the protein hydrolysates may have antioxidant and functional activity. However, the effects of such components along with the protein concentrate on antioxidant and antibacterial properties (crude protein) were evaluated. Therefore, the effects of these components can be considered constant in all hydrolyzed products. In this study, the effects of the type of proteases on the degree of hydrolysis and antioxidant indices of primary protein were investigated although proteases do not have much effect on polysaccharides due to their specific activity. Hence, the changes in these properties after enzymatic hydrolysis can be neglected.

### 3.6 Morphologic observations

**FIGURE 4:**
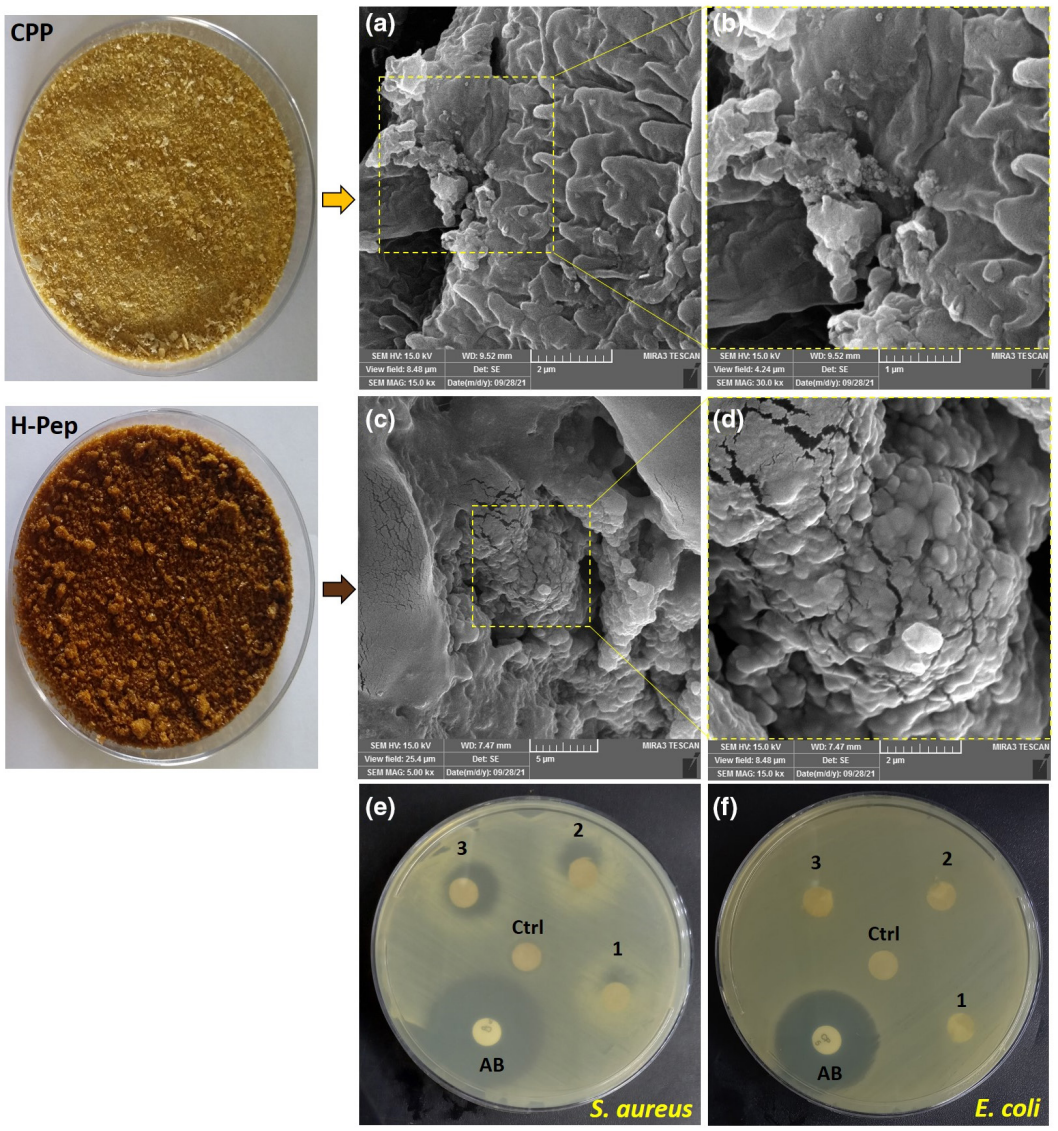
(a–d) Morphology and surface structure of corn pollen protein (CPP) before and after enzymatic hydrolysis by pepsin (H‐Pep). (a,b) SEM images of CPP; (c,d) SEM images of H‐Pep hydrolysate at two magnifications. (e,f) Antibacterial effect of corn pollen protein (CPP) before and after enzymatic hydrolysis by pepsin (H‐Pep) against *S. aureus* and *E. coli*. AB: Antibiotic (ciprofloxacin) disk; Ctrl: control disk containing CPP; 1, 2, and 3: H‐Pep hydrolysate disks at concentrations of 50, 100, and 200 mg mL^−1^, respectively.

The overall appearance of corn pollen protein before and after hydrolysis by pepsin and SEM images of CPP and pepsin hydrolyzed protein (H‐Pep) is shown in Figure 4a–d. SEM showed aggregated particles with different sizes in both samples. However, CPP particles were smooth on their surface, whereas enzymatic hydrolysis led to the appearance of a rough surface with more cracks/cavities in the hydrolyzed sample. Based on the results of HPLC and the degree of initial protein hydrolysis, it can be concluded that the structural changes after hydrolysis were due to enzyme activity and degradation of protein units.

### 3.7 Antibacterial properties

In previous sections, it was shown by DH (Figure 1a) and SDS‐PAGE (Figure 1b) that pepsin was more effective in hydrolyzing primary CPP compared to other two enzymes used. Moreover, the pepsin hydrolyzed sample (H‐Pep) had more antioxidant peptides (Figure 2d) and provided the most effective free radical scavenging activity and antioxidant properties (Figure 3) compared to those of other hydrolysates (H‐Pan and H‐Tri). Therefore, antibacterial effect of only H‐Pep sample was studied here. Figure 4e,f demonstrates disk diffusion tests for Gram‐positive (*S. aureus*) and Gram‐negative (*E. coli*) bacteria. Antibiotic disk of ciprofloxacin was used as positive control for both bacteria (Aghamirzaei et al., 2015). The antibacterial activity was assessed by evaluating the diameter of inhibition zone (DIZ) (Peighambardoust et al., 2016, 2022). CPP showed no inhibition zones for both bacteria. H‐Pep at concentrations used (50, 100, and 200 mg mL^−1^ for disks 1, 2, and 3, respectively) did not show antibacterial effect against *E. coli*, whereas a significant dose‐dependent antibacterial effect was observed for *S. aureus* with DIZ values of 15, 21, and 25 mm in disks 1, 2, and 3, respectively. This difference is related to the existence of peptidoglycan cell walls in Gram‐negative bacteria (*E. coli*), which itself is surrounded by an outer membrane containing lipopolysaccharide. This makes *E. coli* more resistant to antibacterial effect of peptides, as can be seen in Figure 4f, where no inhibition zones were seen. These results are in accordance with several works reporting that Gram‐negative bacteria are more resistant than Gram‐positive bacteria (Jemil et al., 2014).

### 3.8 Cytotoxic activity

**FIGURE 5:**
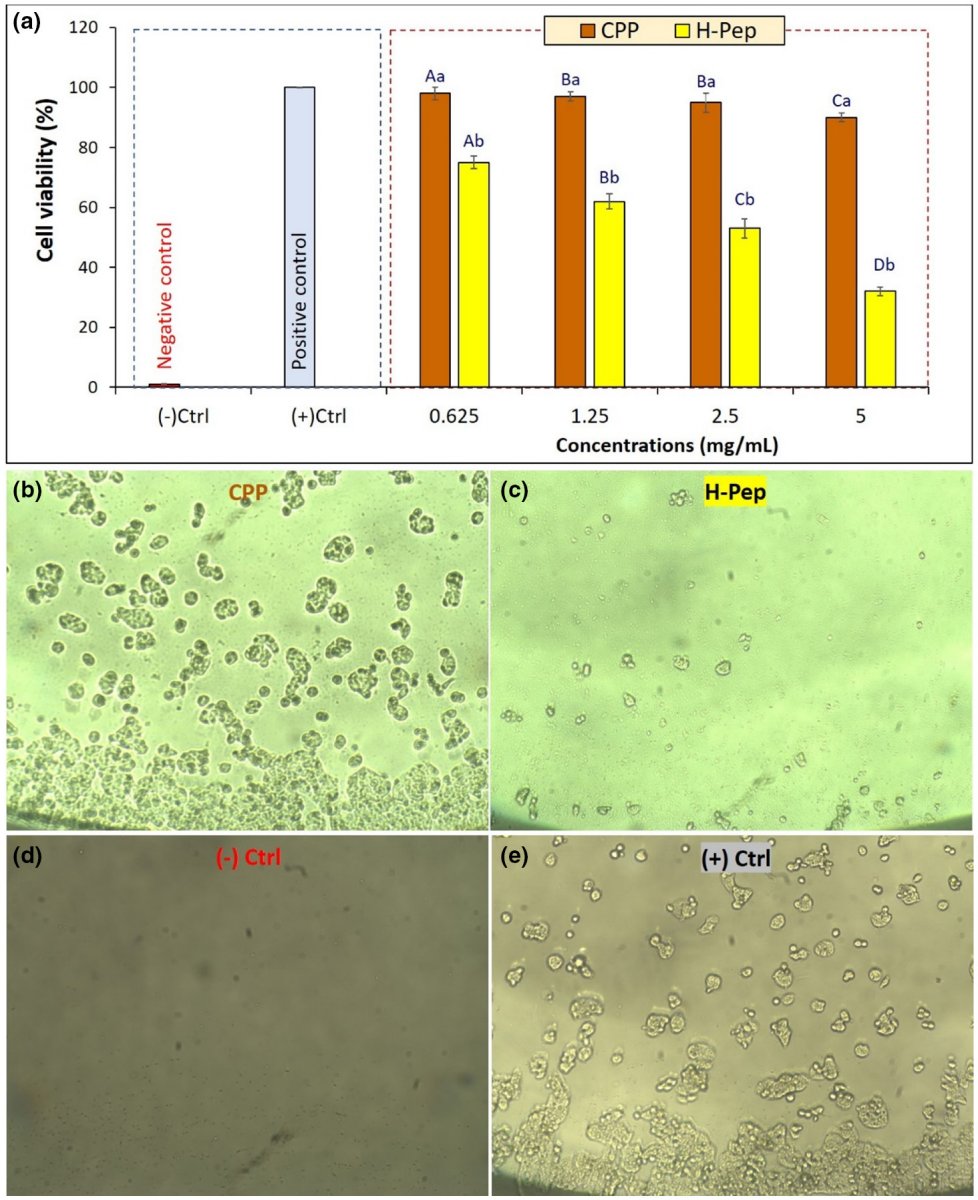
(a) The viability percentage of HT‐29 colon cancer cell line against different concentrations of CPP and H‐Pep hydrolysate after 24 h incubation time. Data are the mean of triplicate measurements. Error bars represent SD values; different lower‐case and upper‐case letters indicate significant (*p* < .05) differences between concentrations and treatments, respectively. (b–e): Representative optical microscopic images (50× magnification) of HT‐29 cells in CPP (a), H‐Pep (b), negative control (d), and positive control (e) are shown.

In this study, MTT test was used to study the effect of H‐Pep hydrolysate on colon cancer cell line (HT‐29). The MTT test determined the amount of cell activity by reducing the yellow color and the formation of insoluble formazan crystals. As shown previously, pepsin effectively hydrolyzed primary CPP compared to other enzymes. Moreover, pepsin hydrolyzed sample (H‐Pep) had more antioxidant peptides (Figure 2d), and provided the most effective free radical scavenging activity and antioxidant properties (Figure 3) as compared with other hydrolysates (H‐Pan and H‐Tri). Thus, H‐Pep was used in MTT assay. Amino acid composition results confirmed the presence of more free functional peptides in the H‐Pep hydrolyzed sample, providing the possibility of their anticancer effect as shown in MTT study. Figure 5a demonstrates the effect of exposure of CPP and H‐Pep at different concentrations on the proliferation of HT‐29 cancer cells.

These results showed that CPP, as a nonhydrolyzed protein, did not generally affect cell proliferation, except at the highest concentrations of 5 mg mL^−1^, a 10% reduction was observed. However, hydrolyzed sample by pepsin (H‐Pep) significantly (*p* < .05) reduced the viability of cancer cells in a concentration‐dependent manner compared to the primary protein (CPP). For example, increasing exposure concentrations of peptides led to significant decrease in the cells' viability. The lowest cell viability of 32 ± 2.8% was obtained for H‐Pep at a concentration of 5 mg mL^−1^. Figure 5b,c demonstrates representative optical microscope images of HT‐29 cancer cells exposed to CPP and H‐Pep hydrolysate (at a concentration of 2.5 mg mL^−1^). Comparing the microscopic images of CPP and H‐Pep clearly show that there is a substantial reduction in cells in the presence of H‐Pep sample. Images of negative and positive controls are presented for a matter of comparison (Figure 5d,e), where, in fresh MEM growth media (negative control), there was no cell growth, and in SDS‐included medium (positive control), complete cell growth was observed. Our results are in agreement with the report by Umayaparvathi et al. (2014) who showed the time‐ and dose‐dependent effect of oyster protein hydrolysates against HT‐29 colon cancer cells. Similarly, a dose‐dependent increase in the cytotoxicity of protease XXIII (PA) digests of tuna cooking juice was observed (Hung et al., 2014). Karami, Peighambardoust, Hesari, Akbari‐Adergani, and Andreu (2019a) also reported that wheat germ protein hydrolysates obtained by alcalase, pepsin, or proteinase K had significant antioxidant activities and the ability to decrease A549 cell viability in a concentration‐dependent manner. In a very recent study, anticancer activity and apoptotic potential of pepsin and pancreatin polypeptides from chia (*Salvia hispanica*) protein (Quintal‐Bojórquez et al., 2021) and pepsin hydrolysates from *Amaranthus cruentus* protein (Ramkisson et al., 2020) were proven.

## 4 CONCLUSION

Bioactive peptides are considered effective antioxidants with health‐promoting properties and could prolong food shelf‐life beyond their basic nutritional value. This study aimed to evaluate physicochemical, antioxidant, antimicrobial, antiproliferative, and in vitro cytotoxic properties of corn pollen protein hydrolysates obtained by different enzymes. Proteolytic activity in terms of degree of hydrolysis and SDS‐PAGE analysis was measured in all hydrolysates. The degree of hydrolysis and SDS‐PAGE analysis revealed higher proteolytic activity of pepsin compared to the other two enzymes used in this study. The antioxidant capacity of hydrolysates was evaluated using Trolox equivalent, DPPH and hydroxyl radical scavenging activities, chelation of iron and copper metals, and total antioxidant activity. The enzymatic hydrolysis using pepsin, trypsin, and pancreatin resulted in polypeptides with strong antioxidant properties such as free radicals scavenging, metal chelating, antimicrobial activity against Gram‐positive bacteria, and a noticeable antiproliferative effect on HT‐29 colon cancer cell line. Antioxidant activity was found to be affected by the type of enzyme and the concentration of hydrolysates. Functional amino acids such as antioxidant types were most predominant in pepsin‐treated samples. We showed that these activities were dependent on the type of peptidase and the concentration of hydrolysates used. Among the enzymes investigated, pepsin exhibited the most enhanced biological activity and functional properties followed by pancreatin and trypsin. The combined impact of antioxidant, antibacterial, and anticancer properties of hydrolysates from corn pollen protein suggested the way to the potential application of these polypeptides as nutraceuticals for functional foods with health‐promoting effects. Of course, more studies are required to investigate the structural properties, other health benefits, effects of other enzymes, and also in vivo analysis of these polypeptides in animal models.

## CONFLICT OF INTEREST STATEMENT

The authors confirm that they have no conflicts of interest with respect to the work described in this manuscript.

## Data Availability

Data will be available on request.
